# Finnish Palliative Care Nurses’ and Physicians’ Perceptions of Spirituality and Spiritual Care Related to Their Attitudes toward End-of-Life Care

**DOI:** 10.1089/pmr.2023.0078

**Published:** 2024-07-13

**Authors:** Raimo Goyarrola, Annamarja Lamminmäki, Virpi Sipola, Ikali Karvinen, Minna Peake, Suvi-Maria Saarelainen, Nina Santavirta, Leila Niemi-Murola, Reino Pöyhiä

**Affiliations:** ^1^ School of Medicine, University of Eastern Finland, Kuopio, Finland.; ^2^ Department of Oncology, University Hospital, Kuopio, Finland.; ^3^ Evangelical-Lutheran Church of Finland, Helsinki, Finland.; ^4^ Faculty of Health & Finn Church Aid (FCA), University of Eastern Finland, Kuopio, Finland.; ^5^ Palliative Care Center Siun Sote, University of Eastern Finland, Joensuu, Finland.; ^6^ School of Theology, Philosophical Faculty, University of Eastern Finland, Joensuu, Finland.; ^7^ Faculty of Educational Sciences, University of Helsinki, Finland.; ^8^ Department of Anesthesiology and Intensive Care Medicine, Helsinki University Hospital, University of Helsinki (Clinicum), Helsinki, Finland.

**Keywords:** attitudes, end-of-life care, nurses, palliative care, physicians, questionnaires, spiritual care, spirituality, surveys

## Abstract

**Background:**

Spiritual care constitutes an indispensable aspect of palliative care (PC). Health care professionals encounter challenges when addressing spiritual care at the end of life. Developing appropriate attitudes toward end-of-life care can facilitate the acquisition of competencies needed for effective delivery of spiritual care.

**Aim:**

To explore the perceptions of spiritual care and attitudes toward end-of-life care among PC professionals.

**Design:**

The Finnish version of the “Spirituality and Spiritual Care Rating Scale” (SSCRS-FIN) and a newly developed “Attitudes toward End-of-Life Issues” (AEOLI) questionnaire were validated and utilized.

**Setting/Participants:**

Both questionnaires were distributed to PC professionals involved in PC through an online survey. Exploratory and confirmatory factor analyses were conducted. The newly derived factors were subsequently examined for their associations with age, gender, profession, affiliation with a religious community, personal interpretation of spirituality, and years of professional experience.

**Results:**

A total of 204 participants took part in the study (163 nurses, 19 nursing students, and 22 physicians). Exploratory factor analysis demonstrated satisfactory internal consistency, as indicated by Cronbach’s alpha coefficients, for the five factors of SSCRS-FIN: “Spirituality” (0.733), “Existential” (0.614), “Spiritual Needs” (0.599), “Passive Spiritual Care” (0.750), and “Active Spiritual Care” (0.665); and for the seven factors of AEOLI: “Anxiety” (0.823), “Discussion” (0.924), “End-of-Life” (0.573), “Education” (0.692), “Medically Induced Death” (0.859), “Suffering” (0.671), and “Knowledge” (0.444). Confirmatory factor analysis demonstrated satisfactory fit values for both questionnaires. Significant positive correlations were observed between end-of-life care and the factors “Existential,” “Spiritual Needs,” and spiritual care factors, whereas an inverse correlation was found among “Anxiety,” “Medically Induced Death,” and all factors of SSCRS-FIN.

**Conclusions:**

Valid and reliable questionnaires for assessing spiritual care (SSCRS-FIN) and attitudes toward end-of-life care (AEOLI) were developed. Attitudes toward end-of-life care were positively correlated with perceptions of spiritual care.

## Key Message

Attitudes toward spiritual care and spirituality are related to, but not entirely synonymous with, attitudes toward end-of-life care; greater focus on spiritual care is associated with increased willingness to engage in end-of-life discussions, heightened readiness to provide support during the dying process, and reduced distress among palliative care professionals.

## Background

Spiritual care (SC) entails acknowledging, respecting, and addressing the spiritual needs of patients,^1^ particularly in the context of illness and crises.^2,3^ Various forms of SC have been demonstrated to enhance patients’ quality of life,^4–8^ and improve their end-of-life (EOL) care.^9,10^ Neglecting to provide SC can result in depression, diminished physical wellbeing, and spiritual distress.^11^ However, only 6% to 28% of palliative patients actually receive the necessary SC they require.^12,13^

SC remains insufficiently integrated into palliative care (PC) for various reasons.^14,15^ Patients and their loved ones may find it challenging to recognize and express their spiritual needs, as these are deeply personal and intimate.^16^ Health care professionals may lack the time and resources due to excessive workloads,^17–20^ insufficient knowledge and skills,^21,22^ and other difficulties in recognizing and alleviating their patients’ spiritual distress.^23^

While it is recommended that health care professionals in PC actively practice SC, there is still limited understanding of how they perceive it.^24,25^ SC necessitates multiprofessional teams to address the various types of spiritual needs that arise in care.^3,13^ However, it remains unclear whether SC is considered by nurses and physicians to be an integral part of EOL care or a separate aspect managed solely by spiritual counselors.^26^ Is SC a religious activity, a dimensional issue, or a cultural behavior?^27,28^

Although validated instruments are available for assessing SC,^29^ they have not been widely utilized in conjunction with PC, especially in EOL care. Exploring the attitudes of staff would be essential to enhance the quality and availability of SC.^30–32^ PC professionals’ attitudes and views about SC should be studied concurrently with their attitudes on EOL care issues, a gap that has not been addressed thus far.

## Methods

### Aim

The aim was to examine the attitudes of PC professionals toward SC and EOL care. For this purpose, a Finnish version of the “Spirituality and Spiritual Care Rating Scale” (SSCRS) and a new assessment tool, “Attitudes toward End-of-Life Issues” (AEOLI) were composed and validated.

### Study design and participants

The original English version of the SSCRS was translated into Finnish (see below “Instruments”), and a new instrument, AEOLI, was developed. After piloting and validation, these tools were used to examine attitudes among health care professionals using the *Webropol* program (Webpropol Oy/Ltd, Helsinki).

Participants were recruited from three PC units in Eastern Finland (Mikkeli, Kuopio, and Joensuu), through the webpage of the Finnish Association for Palliative Care, and through personal communications.

A total of 204 participants were included in the final analyses (Table 1), with 176 subjects (155 nurses and 21 doctors) completing the SSCRS-FIN scale and 185 (165 nurses and 20 doctors) completing the AEOLI questionnaire. A subset of 157 participants responded to both questionnaires.

**Table 1.** Participants Characteristics

Variable	Total
*Total participants*	204
*Gender*	
Female	183 (89.7%)
Male	20 (9.8%)
*Age (M / SD)*	
Female	44.81 (10.74)
Male	46.40 (14.22)
*Profession*	
Nurses	163 (79.9%)
Nursing students	19 (9.3%)
Doctors	22 (10.78%)
*Religious community belonging*	
Yes	95 (46.56%)
No	109 (53.43%)
*Importance of spirituality*	
Not at all	23 (11.3%)
A little	41 (20.1 %)
Quite a bit	57 (27.9 %)
A lot	46 (22.5 %)
Very much	37 (18.1 %)
*Experience in palliative care*	
I worked	66 (32.4%)
I currently work	123 (60.3%)
I have no experience	14 (6.9%)
*Time working in palliative care*	
1 to 9 years	117 (57.4%)
10 to 19 years	41 (20.1%)
20 to 29 years	32 (15.7%)
30 to 49 years	14 (4.9%)

M, mean; SD, Standard deviation.

### Instruments

#### a. “Spirituality and spiritual care rating scale” (SSCRS)

The original SSCRS comprises 17 items about nurses’ attitudes toward SC, categorized into four factors on a 5-point Likert scale.^33^ The item 18 regarding physicians’ attitudes was added in the Finnish version, SSCRS-FIN: “I believe spiritual care also belongs to the medical doctor’s work.” The Finnish translation of SSCRS was performed by two independent translators and subsequently back-translated by two other individuals (see supplementary data S1 SSCRS-FIN).

#### b. Questionnaire “attitudes toward end-of-life issues” (AEOLI)

This Finnish questionnaire (see supplementary data S1 AEOLI) consisted of 24 items on a 7-point Likert scale (1 = totally disagree, 4 = neither agree nor disagree, 7 = totally agree). It covered education and knowledge about EOL issues, experiences of distress or suffering when working with dying patients, communication with patients and their families, and a single question about acceptance of euthanasia.

The development and implementation of this questionnaire were based on a 10-year process, which involved testing shorter versions among 218 Finnish health care professionals (unpublished information). The questionnaire was partially based on national recommendations for EOL care^34^ and an English questionnaire.^35^

### Validation procedure

#### Face and content validity

They were assessed by four nurses and four physicians to evaluate clarity, meaningfulness, and the presence of potentially offensive items in the questionnaires.

#### Structural validity–Factor analyses

Quantitative analysis, which included descriptive statistics and factor analysis, was conducted for both questionnaires.^36^ Exploratory factor analysis (EFA) with principal axis factoring and varimax rotation was employed on the combined data from all responses to assess the theoretical relevance^37,38^ of both scales (SSCRS-FIN and AEOLI). The determination of the number of factors was based on both Kaiser’s Criteria (Eigenvalue > 1) and Cattell’s Scree Test. Confirmatory factor analysis (CFA) was performed to assess the goodness of fit of the results to the original structure established in the SSCRS. The expected values for recommended fit indices^39^ were as follows: the ratio of the chi-square statistic to the degrees of freedom (CMIN/DF: <3.0); Comparative fit index (CFI: >0.90 for acceptable fit); root mean squared error of approximation (RMSEA: <0.06 for reasonable fit) with its *p* value of Close-Fit (PCLOSE: >0.05), and standardized root mean square residual (SRMR: <0.08). The maximum likelihood estimation technique was utilized. Internal consistency was assessed using Cronbach’s alpha coefficient.

#### Convergent/divergent and construct validity

The relationships of items with respondents’ age, gender, profession, affiliation with a religious community, personal understanding of spirituality, and years of professional experience were studied for both scales. The correlations between the factors in both scales were also examined.

### Reliability: Test–retest

A two-week test–retest analysis was conducted on the SSCRS-FIN and AEOLI scales with 12 and 6 health professionals, respectively.

### Data collection and statistical methods

Data collection was conducted electronically using the free Webropol application between December 2022 and April 2023. There were no missing data in the SSCRS-FIN scale, and 21 empty answers (0.47%) were found in the AEOLI questionnaire.

The distribution of data was checked with the Kolmogorov–Smirnov test, measures of skewness and kurtosis, and z-values of skewness and kurtosis. Items 5, 11, 12, and 15 for SSCRS-FIN and items 2, 5, 14, 22, and 23 for AEOLI showed a normal distribution. Furthermore, the variables of gender, profession, and working years demonstrated non-normal distributions with z-values of 23.53 for gender, 11.14 for profession, and 6.18 for working years. Mean values with standard deviations were calculated.

Pair-wise comparisons of variables were examined in normally distributed data with paired samples *t-test* and in non-normally distributed data with the Mann–Whitney U test. Kruskal–Wallis’ test was used for multiple associations between scores and categorical variables. Pearson’s correlation coefficients were calculated when appropriate. Statistical significance was set at *p* < 0.05 (*).

Statistical and factor analyses were conducted using IBM SPSS Statistics 27 (Version 27.0.1, Armonk, NY: IBM Corp.) and AMOS 27.0 (Armonk, NY: IBM Corp.) software.

### Ethical considerations

Consultation from ethical aspects to collect data was requested from the University of Eastern Finland Research Ethics Committee. According to Finnish legislation and ethical guidelines, a formal ethical assessment is not required for public, anonymous, and voluntary online surveys that do not collect individual data. Participants were assured that data would be handled exclusively for research purposes and with the utmost confidentiality, and it would not be stored after analysis. In addition, Dr. McSherry granted us permission to translate and utilize the SSCRS scale.

## Results

### Content validity

Most participants (156%–86.2%) found the items of SSCRS-FIN easily understandable. No changes were proposed in translations. The overall ratings for attitudes toward SC ranged from 3.36 to 4.76 on average (Table 2), indicating a widespread understanding and positive views on spirituality and SC. The respondents considered SC as a part of both nurses’ and physicians’ work.

**Table 2.** Exploratory Factor Analysis SSCRS-FIN (Range 1–5)

Factor	Item	Mean	SD	Com	Rot
1. Spirituality Var: 26.82% EV: 4.82 Cronbach’s α: 0.733	(j-10) I believe spirituality is to do with the way one conducts one’s life here and now.	4.09	0.732	0.753	0.803
	(f-6) I believe spirituality is about finding meaning in the good and bad events of life.	4.15	0.786	0.423	0.603
	(l-12) I believe spirituality is a unifying force, which enables one to be at peace with oneself and the world.	4.33	0.654	0.556	0.405
2. Existential Var: 10.55% EV: 1.89 Cronbach’s α: 0.614	(p-16) I believe spirituality does not apply to Atheists or Agnostics.	1.56	0.673	0.386	−0.590
	(r.18) I believe spiritual care also belongs to medical doctor’s work.	4.30	0.837	0.341	0.447
	(m-13) I believe spirituality does not include areas such as art, creativity, and self expression.	1.73	0.736	0.257	−0.439
	(d-4) I believe spirituality involves only going to Church/Place of Worship.	1.30	0.482	0.222	−0.413
	(o-15) I believe spirituality involves personal friendships, relationships.	3.80	0.926	0.365	0.412
3. Spiritual Needs Var: 7.79% EV: 1.40 Cronbach’s α: 0.599	(i-9) I believe spirituality is about having a sense of hope in life.	4.13	0.748	0.413	0.535
	(e-5) I believe spirituality is not concerned with a belief and faith in a God or Supreme being.	2.39	1.020	0.342	−0.499
	(h-8) I believe nurses can provide spiritual care by enabling a patient to find meaning and purpose in their illness.	3.36	1.123	0.260	0.485
	(c-3) I believe spirituality is concerned with a need to forgive and a need to be forgiven.	4.11	0.900	0.253	0.459
	(q-17) I believe spirituality includes people’s morals.	3.94	0.794	0.250	0.394
4. Passive Spiritual Care Var: 6.46% EV: 1.16 Cronbach’s α: 0.750	(k-11) I believe nurses can provide spiritual care by listening to and allowing patients’ time to discuss and explore their fears, anxieties and troubles.	4.58	0.518	0.797	0.788
	(g-7) I believe nurses can provide spiritual care by spending time with a patient giving support and reassurance especially in time of need.	4.41	0.636	0.529	0.497
	(n-14) I believe nurses can provide spiritual care by having respect for privacy, dignity, and religious and cultural beliefs of a patient.	4.66	0.497	0.354	0.414
5. Active Spiritual Care Var: 5.72% EV: 1.03 Cronbach’s α: 0.665	(a-1) I believe nurses can provide spiritual care by arranging a visit by the hospital Chaplain or the patient’s own religious leader if requested.	4.76	0.526	0.572	0.715
	(b-2) I believe nurses can provide spiritual care by showing kindness, concern, and cheerfulness when giving care.	4.59	0.645	0.527	0.666

Com, communalities; EV, eigenvalue; M, mean; Rot, varimax rotated factor matrix; SD, standard deviation; Var, variance.

A total of 170 individuals (91.9%) found the AEOLI questionnaire understandable. Overall, the attitudes toward EOL care were predominantly positive and compassionate (Table 3).

**Table 3.** Exploratory Factor Analysis APCD (Range 1–7)

Factor	Item	Mean	SD	Com	Rot
1. Anxiety Var: 18.50% EV: 4.44 Cronbach’s α: 0.823	3. The thought of encountering the sorrow of a dying patient feels distressing.	2.98	1.529	0.818	0.882
	2. Facing suffering feels distressing.	3.72	1.737	0.666	0.719
	1. Encountering dying people feels distressing.	2.18	1.214	0.588	0.705
2. Discussion Var: 9.42% EV: 2.26 Cronbach’s α: 0.924	18. I find it important to discuss the DNR (Do No Resuscitate) decision with the patient’s family members beforehand.	5.88	1.178	0.901	0.920
	17. I find it important to discuss the DNR (Do No Resuscitate) decision with the patient beforehand.	6.00	1.166	0.832	0.906
3. End-of-Life Var: 8.55% EV: 2.05 Cronbach’s α: 0.573	9. Respecting the patient’s religious beliefs convictions is an essential part of end-of-life care.	6.88	0.362	0.415	0.564
	7. Doctor/nurse should support the patient preparing to die.	6.91	0.301	0.331	0.548
	8. Depression of a patient in end-of-life care can be treated.	6.25	0.88	0.293	0.478
	10. Reflection upon spiritual issues is an essential part of end-of-life care.	6.31	0.966	0.314	0.494
	6. Physicians/nurses have a responsibility to provide bereavement care to the patient’s family members after death.	6.88	0.426	0.326	0.489
4. Education Var: 7.65% EV: 1.83 Cronbach’s α: 0.692	14. I am satisfied with the education I have received about end-of-life care during my training up to now.	5.11	1.503	0.631	0.788
	13. I believe that my education has given me/will give me the competence needed to alleviate the patients’ suffering.	5.53	1.426	0.581	0.732
	12. I believe that I have received the competence to treat a dying patient.	5.90	1.185	0.527	0.471
	21. I am familiar with the term end-of-life care.	6.79	584	0.226	0.297
5. Medically Induced Death Var: 6.18% EV: 1.48 Cronbach’s α: 0.859	22. Euthanasia should be legalized in Finland.	4.29	2.008	0.967	0.956
	23. Doctors should be able to assist their patients’ commit suicide.	3.55	1.835	0.613	0.769
6. Suffering Var: 6.04% EV: 1.45 Cronbach’s α: 0.671	15. Psychological suffering can be as severe as physical suffering	6.78	0.454	0.870	0.908
	16. Social unease can be as stressful as physical fatigue suffering.	6.61	0.644	0.692	0.774
	11. A patient has a right to refuse from medical treatment.	6.73	0.601	0.225	0.231
7. Knowledge Var: 5.28% EV: 1.26 Cronbach’s α: 0.444	20. End-of-life care is provided only for patients suffering from cancer.	1.21	0.444	0.455	0.602
	19. End-of-life care means giving up all medical treatment.	1.56	0.937	0.366	0.599
	24. I know who to contact for consultation in palliative care.	5.63	1.576	0.431	−.0536
	5. I would like to discuss facing death with a more experienced colleague.	3.86	1.923	0.277	0.264
	4. I try to avoid participating in the decisions regarding ending curative care.	2.12	1.368	0.214	0.234

Com, communalities; EV, eigenvalue; M, mean; Rot, varimax rotated factor matrix; SD, standard deviation; Var, variance.

### Structural validity

#### a. SSCRS-FIN

The values of the Kaiser–Meyer–Olkin Measure of Sampling Adequacy (0.793) and Bartlett’s Test of Sphericity (*p* value: 0.000) indicated that the data were suitable for factor analysis. The communalities of the items ranged from 0.394 (item 17) to 0.803 (item 10). The analysis of the 18 items resulted in a five-factor model (Table 2).

These factors accounted for 57.34% of the variance in the scores. The identified factors were labeled based on the content and meaning of the items: Factor 1 “Spirituality” (items 10, 6, and 12); Factor 2 “Existential” (items 16, 18, 13, 4, and 15); Factor 3 “Spiritual Needs” (items 9, 5, 8, 3, and 17); Factor 4 “Passive Spiritual Care” (items 11, 7, and 14); and Factor 5 “Active Spiritual Care” (items 1 and 2).

In CFA, the fit indices were as follows: the ratio of the chi-square statistic to the degrees of freedom (CMIN/DF: 187.469/121) = 1.549; CFI = 0.912; RMSEA = 0.056; PCLOSE = 0.255, and SRMR = 0.064.

The standardized estimates of the five-factor model can be seen in Supplementary Figure S1.

### Internal consistency

The alpha values for the five separate factors were found acceptable as follows: Spirituality (0.733), Existential (0.614), Spiritual Needs (0.599), Passive Spiritual Care (0.750), and Active Spiritual Care (0.665).

To examine the impact of adding item 18 on the EFA results, we conducted separate EFA for both the 17-item and 18-item versions. Both analyses yielded five factors, and interestingly, the 18-item version exhibited a slightly higher Cronbach’s alpha (0.804) compared with the 17-item version (0.790). These findings suggest that both questionnaires demonstrate comparable validity and reliability.

#### b. AEOLI

The sampling adequacy measure yielded a value of 0.668, indicating satisfactory sampling adequacy. The sphericity condition was also satisfied (*p* value = 0.000). The communalities of the items ranged from 0.231 (item 11) to 0.956 (item 22).

Initially, an eight-factor model was obtained from the analysis of the 24 items. However, upon reviewing the item distribution, we decided to consolidate it into a more parsimonious seven-factor model (Table 3). These seven factors accounted for 61.64% of the variance in the scores and were named based on the concepts contained in the respective items: Factor 1 “Anxiety” (feeling distressing)—items 3, 2, and 1; Factor 2 “Discussion” (focus on: “Do Not Resuscitate”)—items 18 and 17; Factor 3 “End-of-Life” (mental and spiritual issues also after death)—items 9, 7, 8, 10, and 6; Factor 4 “Education” (training and competence)—items 14, 13, 12, and 21; Factor 5 “Medically Induced Death” (euthanasia and physician assisted suicide)—items 22 and 23; Factor 6 “Suffering” (psychological, social)—items 15 and 16; and Factor 7 “Knowledge” (concepts, advice)—items 20, 19, 24, 5, and 4.

In CFA, the fit indices were as follows: the ratio of chi-square statistic to the degrees of freedom (CMIN/DF: 315.340/228) = 1.383; CFI = 0.934; RMSEA = 0.046; PCLOSE = 0.717, and SRMR = 0.063.

The standardized estimates of the seven-factor model can be seen in Supplementary Figure S2.

### Internal consistency

The Cronbach’s alpha values were: “Anxiety” (0.823), “Discussion” (0.924), “End-of-Life” (0.573), “Education” (0.692), Medically Induced Death (0.859), “Suffering” (0.671), and "Knowledge" (0.444).

### Convergent/divergent and construct validity of SSCRS-FIN and AEOLI

The relationship between the factors and the responders’ individual variables is presented separately for both scales in Table 4. In SSCRS-FIN, statistically significant associations were found between individual spirituality and all spiritual factors, as well as religious belonging with spiritual needs. Physicians expressed slightly more positive attitudes toward the SC of their patients compared with nurses.

**Table 4.** Means, Correlations, and Associations between Variables and Factors

	Age^a^	Gender^b^		Profession^c^			Religious^b^		Spirituality^c^					Experience^c^			Years working^c^			
	44.9	F	M	Nur	NSt	Doc	Yes	No	0	1	2	3	4	Past	Now	No	1–9	10–19	20–29	30–49
SSCRS-FIN (range 1–5)																				
N	176	157	18	138	17	21	88	88	20	35	51	35	35	57	106	12	97	40	28	11
Spi	4.18	4.19	4.09	4.16	4.35	4.22	4.23	4.14	4.07	3.92*	4.21*	4.36*	4.30	4.20	4.18	4.19	4.20	4.19	4.18	4.12
Ext	4.3*	4.30	4.27	4.30	4.42	4.24	4.30	4.3	4.13	4.14*	4.44*	4.29*	4.37	4.29	4.33	4.15	4.28	4.26	4.43	4.33
SN	3.83*	3.79	4.10	3.78*^a^	3.93	4.04*a	4.00**	3.65**	3.52**ab	3.62*cd	3.8*e	3.88*acf	4.21**bdef	3.88	3.79	3.93	3.84	3.78	3.79	3.98
PSC	4.55	4.55	4.52	4.54	4.63	4.59	4.53	4.57	4.45*	4.38*abc	4.59*a	5.65*c	4.62*b	4.61	4.54	4.39	4.56	4.58	4.48	4.58
ASC	−4.67	4.67	4.61	4.67	4.59	4.71	4.69	4.64	4.57	4.47*abc	4.70*a	4.71*b	4.82**c	4.67	4.68	4.62	4.65	4.67	4.75	4.59
AEOLI (Range 1–7)																				
N	185	167	17	148	17	20	86	99	22	39	53	41	30	59	112	13	105	38	31	11
Anx	−2.95 **	2.97	2.88	2.96	3.17	2.71	2.90	3.00	3.36	3.22	2.96	2.63	2.74	3.00**a	2.73**b	4.61**ab	3.09	2.73	2.90	2.60
Disc	5.94	5.93	6.32	5.84*a	6.14	6.47*a	6.08	5.81	5.65*a	5.58*b	6	5.96*	6.45*ab	6.03	5.89	5.84	5.91	5.96	5.91	6.18
EoL	6.64	6.65	6.61	6.66	6.54	6.61	6.67	6.62	6.57	6.54	6.67	6.68	6.72	6.65	6.64	6.66	6.59	6.67	6.71	6.83
Edu	5.83*	5.83	5.76	5.84	5.82	5.75	5.87	5.79	5.50	5.93	5.75	5.98	5.87	5.77**a	5.97**b	4.92**ab	5.8	5.98	5.72	5.9
MID	−3.91 **	4.01*	2.76*	4.11**a	4.41**b	2.05**ab	3.61*	4.18*	4.34**a	4.87**bc	4.22**d	3.65**ce	2.18**abde	4	3.82	4.11	4.13	3.6	3.56	3.9
Suff	−6.70	6.70	6.70	6.70	6.62	6.76	6.74	6.67	6.75	6.62	6.67	6.75	6.74	6.64	6.73	6.71	6.70	6.71	6.65	6.75
Know	−2.26	2.26	2.29	2.20**a	2.89**ab	2.18**b	2.31	2.21	2.32	2.29	2.01**ab	2.33**a	2.50**b	2.35*ac	2.09*ab	3.09**bc	2.38*ab	2.08*b	2.18	1.89*a

N: Number.

*Factors*: Spi, spirituality; Ext, existential; SN, spiritual needs; PSC, passive spiritual Care; ASC, active spiritual care; Anx, anxiety; Disc, discussion; EoL, end-of-life; Edu, education; MID, medically induced death; Suff, suffering; know, knowledge.

*Variables:* Gender (F, female; M, male); Profession (Nur, nurse; NSt, nurse student; Doc, medical doctor); Religious community (Yes/No); Importance of spirituality (0, Not at all; 1, a little; 2, Quiet a bit; 3, A lot; 4, very much); Experience (Past, I have worked in a unit where my duties included palliative care and/or hospice care; Now, I currently work in a unit where my duties include palliative care and/or hospice care; No, I have no experience in palliative care and/or hospice care); Years working in palliative care.

^a^ Pearson’s correlation: (-): negative correlation.

^b^ Independent-Samples Mann–Whitney U Test.

^c^ Independent-Samples Kruskal–Wallis Test.

^*^ Statistically significant differences at 0.05; ** at 0.01 level. a.b.c.d.e: reciprocate associations.

In AEOLI, negative correlations were found between age and “Anxiety” and “Medically Induced Death.” Significant differences in responses to the “Medically Induced Death” factor were observed based on age, gender, profession, religious belonging, and personal spirituality. A positive correlation was observed between age and the factors “Existential,” “Spiritual Needs,” and “Education.” Differences in work experience in PC were reflected significantly in the factors “Anxiety,” “Education,” and “Knowledge,” whereas the number of years worked was positively correlated with the “Knowledge” factor.

Table 5 presents the correlations between SSCRS and AEOLI. Statistically significant positive correlations were found between the factors “End-of-Life care” in AEOLI and “Existential,” “Spiritual Needs,” and “Active and Passive Spiritual Care” factors in SSCRS. “Suffering” correlated positively with all spiritual factors, most strongly with “Spirituality” and “Passive Spiritual Care.” Negative correlations were observed between spiritual care factors and “Anxiety” and “Medically Induced Death.”

**Table 5.** Correlations between the Factors of SSCRS-FIN and AEOLI

FACTORS	Spi	Ext	SN	PSC	ASC
Anx	−0.071	−0.119	−0.144	−0.208^**^	−0.186^*^
Dis	0.170^*^	0.118	0.181^*^	0.132	0.095
EoL	0.137	0.218^**^	0.288^**^	0.294^**^	0.213^**^
Edu	0.100	0.060	0.088	0.123	0.069
MID	−0.098	−0.026	−0.354^**^	−0.081	−0.154
Suff	0.321^**^	0.284^**^	0.175^*^	0.277^**^	0.196^*^
Know	−0.086	−0.196^*^	0.016	−0.230^**^	−0.185^*^

^*^ Correlation is significant at the 0.05 level (2-tailed).

^**^ Correlation is significant at the 0.01 level (2-tailed).

### Reliability: Test–retest

No statistically significant differences were observed between the two measurement times, indicating good stability and reliability of the two questionnaires over time.

## Discussion

### Main findings

The key findings of this study, demonstrated through carefully validated questionnaires, indicate that PC professionals view SC as an integral aspect of their work and exhibit compassionate and positive attitudes toward EOL care. The responders’ understanding of SC was broad and not solely associated with religion. The importance of communication, existential issues, and psychosocial support-related attitudes were recognized in AEOLI, and they correlated positively with those of SC.

Our findings align with recent research, showing that nurses and physicians consider SC as an important part of EOL care and their work.^20,40–43^ In addition, the attitudes of EOL issues were consistent with previous studies.^44,45^

An interesting finding was a negative correlation between the responders’ experienced anxiety and their attitudes toward SC. A positive attitude toward SC may reduce nurses’ and physicians’ personal anxiety in EOL care situations, which is consistent with similar conclusions drawn in previous studies exploring the role of SC alongside PC.^46,47^ Another research study has shown that SC received by patients reduced anxiety in their family members.^48^

However, attitudes and experiences related to PC “Education” did not significantly correlate with SC attitudes, most likely indicating that the received PC education lacked SC components. Indeed, in Finnish nursing and medical schools, SC is not included in the curricula.

In this study, nurses had lower mean values than doctors in the factors of “Spiritual Needs” and “Discussion” in SSCRS and AEOLI, respectively. Previous studies have suggested that nurses who hold negative attitudes toward death may encounter challenges in providing spiritual care^49,50^ and may hesitate to engage in conversations about death.^51^

### Perceptions of the concepts of spirituality and spiritual care (SSCRS-FIN)

Unlike other validated instruments, such as the “Spirituality Assessment Scale”^52^ and the “Spirituality Measurement Scale,”^53^ SSCRS measures attitudes and distinguishes between religiosity and spirituality. The validity, reliability, and feasibility of SSCRS have been demonstrated in various languages and specialties.^54–56^

During the EFA of SSCRS-FIN, a five-factor model appeared to be the best fit. Compared with the original questionnaire, we renamed three factors to better represent the content and meaning of the individual items,^57,58^ and to ensure cultural harmonization. Similar adaptations have been made in the Polish translation of SSCRS.^54^

The internal consistency of the SSCRS-FIN scale (Cronbach’s alpha coefficient: 0.804) was higher compared with the original English questionnaire (0.64),^33^ but within the same range as in other language versions (ranging from 0.7 to 0.9).^54,55,59–61^ The added item asking if SC would also be a part of a physician’s work received high rankings from all respondents. Although we did not comprehensively examine a physician’s role, our findings are in concordance with previous studies.^40,41^

We did not find significant differences between female and male participants in the scores of SC items, contrary to previous research.^62^ Belonging to a religious community was not as significant in the perception of spirituality or the provision of SC as expected, but personal perception of spirituality was. Spirituality is not exclusively associated with religion.^57^ Contrary to a previous study,^63^ we did not observe a significant association between longer work experience and attitudes toward SC or spirituality. Clearly, without education and knowledge of SC, work experience alone is insufficient.^19,57,64–66^

### *Attitudes toward end-of-life* *issues (AEOLI)*

Several tools are available for assessing PC professionals’ views on EOL care.^67,68^ They have mainly been used to detect PC skills and personal attitudes toward death among nurses, physicians, and students in various areas of health care, but they are not available in Finnish. Since none of them seemed suitable for examining attitudes, we developed a new instrument.

Although euthanasia and physician-assisted suicide are not PC,^69–73^ we included them under a topic “Medically Induced Death” in our questionnaire due to the ongoing global debate surrounding EOL care.^74^ Our findings indicated that “Medically Induced Death” negatively correlated with all spiritual factors. Men and physicians were more hesitant toward acceptance of medically induced death than women and nurses, respectively. Individuals with religious affiliation also exhibited more negative attitudes toward “Medically Induced Death” than those without.

### Strengths and limitations

To the best of our knowledge, our study was the first to examine attitudes toward both SC and EOL care among PC nurses and physicians. A strength of our study was the comprehensive examination of the validity and reliability of the tools we used. The relatively large number of responders was recruited from various regions of Finland, and their gender and professional distribution reflect that of Finnish health care professionals. This ensures a reliable picture of the country in our study.

We acknowledge as a limitation that a strict definition of SC was not provided. However, we aimed to examine and rely on the respondents’ personal views. Yet, the Finnish translation of spirituality was included in the questionnaire, encompassing both religiously affiliated spirituality and spirituality broadly linked to transcendent aspects of life.^75^

The AEOLI scale was not compared with previously published ones because they are not available in Finnish. Nevertheless, we propose that such comparisons would be useful, as well as the use of our instrument for professionals other than those in PC.

Finally, another limitation was the low number of physicians and lack of other health care professionals, which reduces the generalizability of our findings to these groups. Only prospective studies would show if professional tailoring of both questionnaires would be needed.

### What this study adds

The study reveals a close relationship between attitudes toward SC and the comprehension of spirituality with attitudes toward EOL care among PC professionals. While these attitudes are closely related, they do not entirely overlap. The SSCRS-FIN and AEOLI questionnaires exhibit high reliability and validity, making them valuable tools for evaluating PC professionals’ attitudes toward SC and EOL care, respectively.

## Conclusions

We have developed valid and reliable Finnish questionnaires, namely the SSCRS-FIN and AEOLI, for measuring attitudes among nurses and physicians toward spirituality/SC and EOL care, respectively. Our study’s findings show that those professionals consider SC an important part of PC irrespective of their religious affiliation. Positive attitudes on SC correlate with those of EOL issues.

## Ethics Approval and Consent to Participate

All methods adhered to the relevant guidelines and regulations. We obtained permission to collect data from the University of Eastern Finland (UEF) Research Ethics Committee (The Finnish National Board on Research Integrity: TENK). Finnish legislation and ethical guidelines do not require formal ethical assessment for public, anonymous, and voluntary online surveys that do not collect individual data. Nevertheless, participants were assured that their personal data would be used exclusively for research purposes, treated with utmost confidentiality, and not stored after analysis. Additionally, Dr. McSherry granted us permission to translate and utilize the SSCRS scale in our study. Each participant provided written informed consent by participating in the online survey.

## Data Availability

The raw data collected during the current study is not publicly available due to state restrictions, as it contains information that could compromise research participant privacy and consent. However, these data are available from the corresponding author upon reasonable request.

## List of abbreviations

AEOLI: Attitudes toward End-of-Life Issues
Anx: Anxiety
ASC: Active Spiritual Care
CFA: Confirmatory factor analysis
CFI: Comparative Fit Index
CMIN/DF: Ratio of chi-square statistic to the degrees of freedom (CMIN/DF)
Disc: Discussion
Doc: Medical Doctor)
EFA: Exploratory factor analysis
Edu: Education
EOL: End-of-life
Experience (Past: I have worked in a unit where my duties included palliative care and/or hospice care
Ext: Existential
*Factors*: SpiSpirituality
Know: Knowledge. *Variables*Gender (FFemale
M: Male)
MID: Medical Induced Death
No: I have no experience in palliative care and/or hospice care
Now: I currently work in a unit where my duties include palliative care and/or hospice care
NSt: Nurse Student
PC: Palliative care
PCLOSE: p-value of Close-Fit
Profession (Nur: Nurse
PSC: Passive Spiritual Care
RMSEA: Root Mean Squared Error of Approximation
SC: Spiritual care
SN: Spiritual Needs
SRMR: Standardized Root Mean Square Residual.
SSCRS: Spirituality and Spiritual Care Rating Scale
Suff: Suffering
