# Supplementary material for: Finnish Palliative Care Nurses’ and Physicians’ Perceptions of Spirituality and Spiritual Care Related to Their Attitudes toward End-of-Life Care
Source: Palliat Med Rep. 2024 Jul 13;5(1):247–57. doi: 10.1089/pmr.2023.0078 (PMC11262586; doi:10.1089/pmr.2023.0078)
Supplement: Supplementary Material [file pmr.2023.0078_Revised_Questionnaires.docx]

**"Spirituality and Spiritual Care Rating Scale" (SSCRS-FIN)**

*ASENTEETKYSELY* 1 = Täysin eri mieltä, 4 = Ei samaa eikä eri mieltä, tai 7 = Täysin samaa mieltä

| 1. Mielestäni hoitajat voivat tarjota spirituaalista (henkistä/hengellistä) hoitoa järjestämällä potilaalle hänen pyynnöstään sairaalapapin tai potilaan oman uskonnon edustajan vierailun. |  |
| --- | --- |
| 1. Mielestäni sairaanhoitajat voivat tarjota henkistä ja hengellistä hoitoa osoittamalla ystävällisyyttä, huolenpitoa ja iloa hoitotyössä. |  |
| 1. Mielestäni spirituaalisuuteen liittyy tarve antaa anteeksi ja saada anteeksi. |  |
| 1. Mielestäni spiritualiteetti tarkoittaa vain kirkossa tai muussa pyhässä paikassa käyntiä. |  |
| 1. Mielestäni spiritualiteettiin ei liity uskoa ja luottamusta Jumalaan tai korkeampaan voimaan. |  |
| 1. Mielestäni spiritualiteetti tarkoittaa merkityksen löytämistä elämän hyvinä ja huonoina aikoina. |  |
| 1. Mielestäni hoitajat voivat tarjota spirituaalista hoitoa viettämällä aikaa potilaan kanssa, tukemalla häntä ja vahvistamalla hänen spirituaalisia tarpeitaan, kun siihen ilmenee tarve. |  |
| 1. Mielestäni sairaanhoitajat voivat tarjota spirituaalista hoitoa mahdollistamalla potilaalle merkityksen ja tarkoituksen löytymisen hänen sairauteensa. |  |
| 1. Mielestäni spiritualiteetti merkitsee toivon kokemista elämässä. |  |
| 1. Mielestäni spiritualiteetti liittyy tapaan, jolla ihminen elää elämäänsä tässä ja nyt. |  |
| 1. Mielestäni sairaanhoitajat voivat tarjota spirituaalista hoitoa kuuntelemalla ja antamalla potilaalle aikaa keskustella peloistaan, ahdistuksistaan ja ongelmistaan ja tutkia/ tarkastella niitä. |  |
| 1. Mielestäni spiritualiteetti on yhdistävä voima, joka mahdollistaa/ antaa mahdollisuuden rauhaan itsen ja maailman kanssa. |  |
| 1. Mielestäni spiritualiteetti ei sisällä taiteen, luovuuden ja itseilmaisun kaltaisia osa-alueita. |  |
| 1. Mielestäni hoitajat voivat tarjota spirituaalista hoitoa kunnioittamalla potilaan yksityisyyttä, arvokkuutta sekä uskonnollisia ja kulttuurisia vakaumuksia. |  |
| 1. Mielestäni spiritualiteetti sisältää henkilökohtaiset ystävyys- ja ihmissuhteet. |  |
| 1. Mielestäni henkisyys ja hengellisyys ei koske ateisteja tai agnostikkoja. |  |
| 1. Mielestäni spiritualiteetti sisältää ihmisten moraalikäsitykset. |  |
| 1. Mielestäni spirituaalinen hoito kuuluu myös lääkärille. |  |

**AEOLI (Attitudes Towards End-of-life Issues)**

(1) Strongly Disagree; (2) Disagree; (3) Somewhat Disagree; (4) Neither Agree Nor Disagree;

(5) Somewhat Agree; (6) Agree; (7) Strongly Agree.

| 1. Encountering dying people feels distressing. |  |
| --- | --- |
| 1. Facing suffering feels distressing. |  |
| 1. The thought of encountering the sorrow of a dying patient feels distressing. |  |
| 1. I try to avoid participating in the decisions regarding ending curative care. |  |
| 1. I would like to discuss facing death with a more experienced colleague. |  |
| 1. Doctor/nurse should support the patient’s loved ones during end-of life´s care and after death. |  |
| 1. Doctor/nurse should support the patient preparing to die. |  |
| 1. Depression of a patient in end-of-life care can be treated. |  |
| 1. Respecting the patient’s convictions is an essential part of palliative care. |  |
| 1. Pondering spiritual issues is part of end-of life care. |  |
| 1. A Patient has the right to refuse treatment. |  |
| 1. I believe that I have received the competence to treat a dying patient. |  |
| 1. I believe that my training has given me/will give me the competence needed to alleviate the patients’ suffering. |  |
| 1. I am satisfied with the knowledge I have received about end-of-life care during my training up to now. |  |
| 1. Mental indisposition can be as stressful as physical suffering. |  |
| 1. Social unease can be as stressful as physical suffering. |  |
| 1. I find it important to discuss the DNR (Do No Resuscitate) decision with the patient beforehand. |  |
| 1. I find it important to discuss the DNR (Do No Resuscitate) decision with the patient’s loved ones beforehand. |  |
| 1. Starting end-of-life care means the ending of all medical treatments. |  |
| 1. End-of-life care is only offered to cancer patients. |  |
| 1. I am familiar with the term end-of-life care. |  |
| 1. Euthanasia should be legalised in Finland. |  |
| 1. Doctors should be able to assist their patients’ commit suicide. |  |
| 1. I know who to contact for consultation in palliative care. |  |

**AEOLI (Attitudes Towards End-of-life Issues)**

*ASENTEETKYSELY* 1 = Täysin eri mieltä, 4 = Ei samaa eikä eri mieltä, tai 7 = Täysin samaa mieltä

| 1. Kuolevan ihmisen kohtaaminen tuntuu ahdistavalta. |  |
| --- | --- |
| 1. Kärsimyksen kohtaaminen tuntuu ahdistavalta. |  |
| 1. Ajatus kuolevan potilaan surun kohtaamisesta tuntuu ahdistavalta. |  |
| 1. Yritän välttää osallistumista parantavasta hoidosta luopumista koskevan päätöksen tekemiseen. |  |
| 1. Haluaisin keskustella kokeneemman työntekijän kanssa kuoleman kohtaamisesta. |  |
| 1. Lääkärin / hoitajan tulee tukea potilaan omaisia saattohoidon aikana ja kuoleman jälkeen. |  |
| 1. Lääkärin / hoitajan tulee tukea potilasta tämän valmistautuessa kuolemaansa. |  |
| 1. Saattohoidossa olevan potilaan masennusta voidaan hoitaa. |  |
| 1. Potilaan vakaumuksen kunnioittaminen on oleellinen osa saattohoitoa. |  |
| 1. Hengellisten asioiden pohtiminen on osa saattohoitoa. |  |
| 1. Potilaalla on oikeus kieltäytyä hoidosta. |  |
| 1. Uskon että olen saanut valmiudet hoitaa kuolevaa ihmistä. |  |
| 1. Uskon, että koulutukseni aikana saan/ olen saanut valmiudet lievittää potilaiden kärsimystä. |  |
| 1. Olen tyytyväinen tähänastisen koulutuksen aikana saamaani saattohoitoa koskevaan tietoon. |  |
| 1. Psyykkinen pahoinvointi saattaa olla yhtä raskasta kuin fyysinen kärsimys. |  |
| 1. Sosiaalinen pahoinvointi saattaa olla yhtä raskasta kuin fyysinen kärsimys. |  |
| 1. Minulle on tärkeää keskustella potilaan kanssa ennen elvytyksestä luopumista koskevan päätöksen tekoa. |  |
| 1. Minulle on tärkeää keskustella omaisten kanssa ennen elvytyksestä luopumista koskevan päätöksen tekoa. |  |
| 1. Saattohoidon aloittaminen merkitsee kaikesta lääketieteellisestä hoidosta luopumista. |  |
| 1. Saattohoitoa annetaan vain syöpäpotilaille. |  |
| 1. Saattohoito on minulle terminä tuttu. |  |
| 1. Eutanasia tulisi laillistaa Suomessa. |  |
| 1. Lääkärin tulee voida avustaa potilastaan itsemurhassa. |  |
| 1. Tiedän konsultaatiotahot palliatiivisessa hoidossa. |  |
