# Supplementary material for: Finnish Palliative Care Nurses’ and Physicians’ Perceptions of Spirituality and Spiritual Care Related to Their Attitudes toward End-of-Life Care
Source: Palliat Med Rep. 2024 Jul 13;5(1):247–57. doi: 10.1089/pmr.2023.0078 (PMC11262586; doi:10.1089/pmr.2023.0078)

**Figure 1**. Confirmatory Factor Analysis (SSCRS-FIN).

The standardized estimates of the five-factor model.


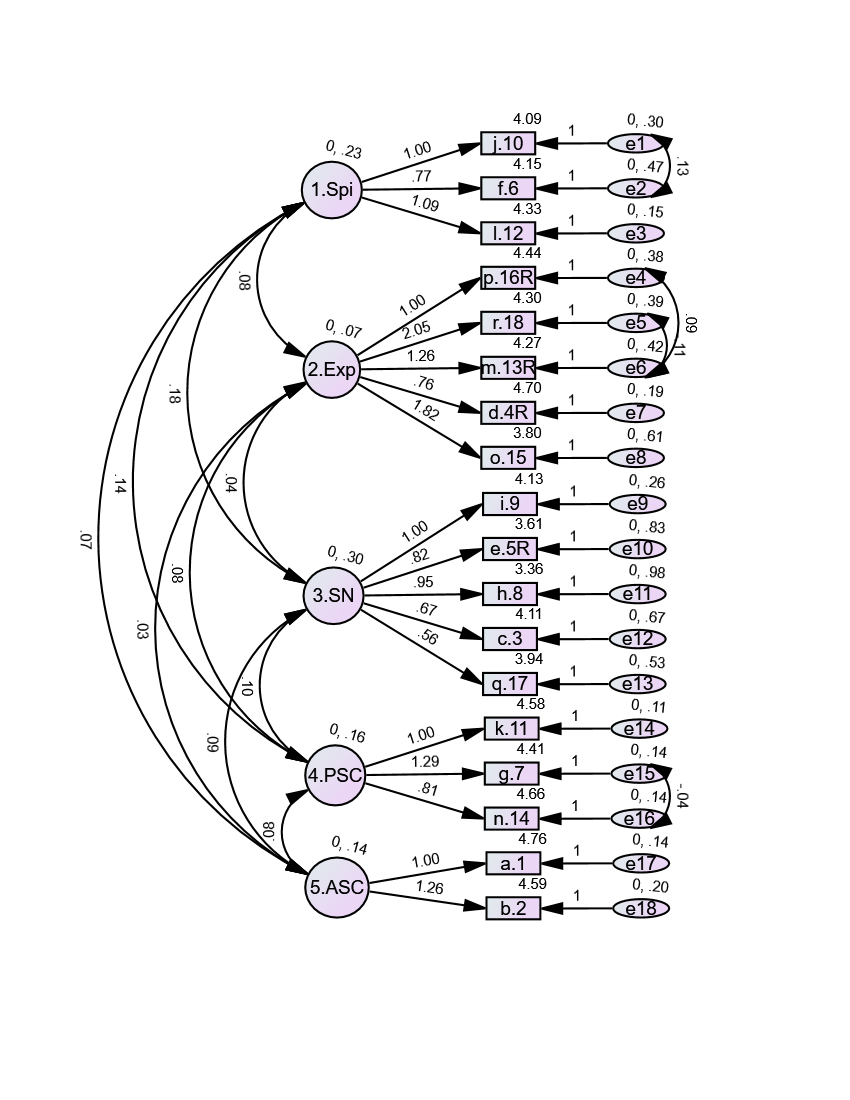


**Figure 2**. Confirmatory Factor Analysis (AEOLI).

The standardized estimates of the seven-factor model.


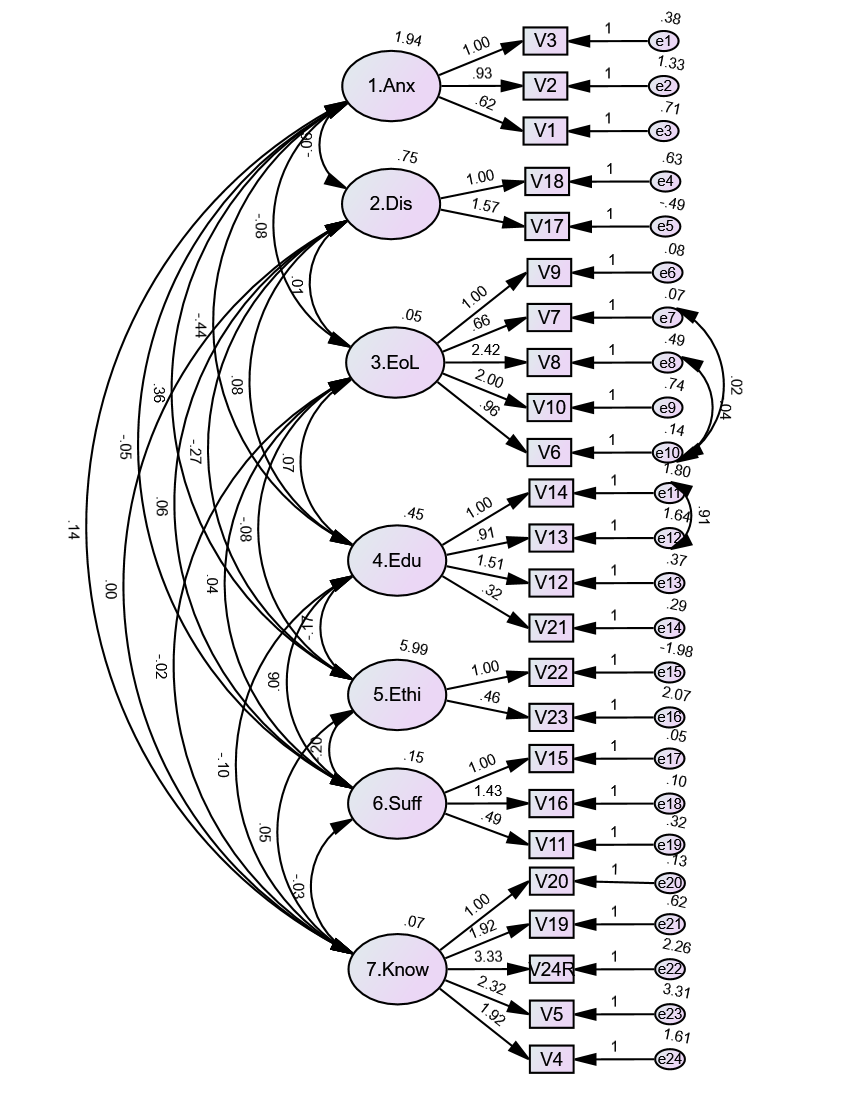

Supplement: Supplementary Figure S1 [file pmr.2023.0078_Revised-Figures.docx]
